# Inhibiting the NLRP3 Inflammasome Activation with MCC950 Ameliorates Diabetic Encephalopathy in db/db Mice

**DOI:** 10.3390/molecules23030522

**Published:** 2018-02-27

**Authors:** Yadong Zhai, Xiangbao Meng, Tianyuan Ye, Weijie Xie, Guibo Sun, Xiaobo Sun

**Affiliations:** 1 Beijing Key Laboratory of Innovative Drug Discovery of Traditional Chinese Medicine (Natural Medicine) and Translational Medicine, Institute of Medicinal Plant Development, Peking Union Medical College and Chinese Academy of Medical Sciences, Beijing 100193, China; zydmailbox@163.com (Y.Z.); xbmeng@implad.ac.cn (X.M.); yetianyuan2013@163.com (T.Y.); ginseng123@163.com (W.X.); 2 Key Laboratory of Bioactive Substances and Resource Utilization of Chinese Herbal Medicine, Ministry of Education, Beijing 100193, China; 3 Key Laboratory of Efficacy Evaluation of Chinese Medicine against Glycolipid Metabolic Disorders, State Administration of Traditional Chinese Medicine, Beijing 100193, China; 4 Zhongguancun Open Laboratory of the Research and Development of Natural Medicine and Health Products, Beijing 100193, China

**Keywords:** diabetic encephalopathy, NLRP3 inflammasome, MCC950, interleukin-1β

## Abstract

Diabetes is associated with a high risk of developing cognitive dysfunction and neuropsychiatric disabilities, and these disease symptomsare termed diabetic encephalopathy (DEP). Inflammation is involved in the development of DEP. The cleavage and maturation of the proinflammatory cytokine interleukin (IL)-1β is regulated by the NLRP3 inflammasome. Obese and type 2 diabetic db/db mice show anxiety- and depression-like behaviors and cognitive disorders associated with hippocampal inflammation. The purpose of this study was to explore the role of NLRP3 inflammasome in DEP. Results showed that expression levels of inflammasome components including NLRP3, apoptosis-associated speck-like protein (ASC), and caspase-1, as well as IL-1β in the hippocampus of diabetic db/db mice were higher than those of non-diabetic db/m mice. Treatment of db/db mice with NLRP3 inflammasome inhibitor MCC950 ameliorated anxiety- and depression-like behaviors as well as cognitive dysfunction, and reversed increased NLRP3, ASC, and IL-1βexpression levels and caspase-1 activity in hippocampus. Moreover, MCC950 treatment significantly improved insulin sensitivity in db/db mice. These results demonstrate that inhibition of NLRP3 inflammasome activation may prove to be a potential therapeutic approach for DEP treatment.

## 1. Introduction

Diabetes mellitus (DM), a chronic metabolic disease, is a serious problem throughout the world. Diabetic encephalopathy (DEP), a diabetic complication, is characterized by cognitive dysfunction and neuropsychiatric disorders [1,2,3]. DM is an independent risk factor for cognitive dysfunction. Several studies have shown that DM is associated with a high risk of vascular dementia and Alzheimer’s disease (AD) [4,5]. In addition, patients with diabetes have a higher risk of suffering from depression than healthy controls [6]. Vascular dysfunction, abnormal glucose metabolism, impaired insulin signaling, and inflammation all contribute to the pathological process of DEP [7,8].

Type 2 diabetes mellitus (T2DM) is a metabolic disease with chronic inflammation [9]. Recent studies highlighted the significance of neuroinflammation as a factor that contributed to the development of neurodegenerative diseases. Evidence has suggested that proinflammatory cytokine interleukin (IL)-1β plays vital roles in diseases related to the central nervous system [10,11,12]. The activation and maturation of IL-1β is mainly regulated by inflammasomes. The nucleotide-binding oligomerization domain-containing protein (NOD)-like receptor family, pyrin domain containing 3 (NLRP3) inflammasome has received widespread attention among the inflammasomes.

The main components of the NLRP3 inflammasome include NLRP3, the adaptor protein apoptosis-associated speck-like protein (ASC), and caspase-1 [13]. Activated caspase-1 can process pro-IL-1β into its mature form IL-1β, which plays vital roles in neuroinflammation. NLRP3 inflammasome activation is implicated in AD, Parkinson’s disease (PD), depression, anxiety, and diabetic complications, and it has been shown that inhibition of NLRP3 inflammasome activation can ameliorate these diseases [14,15,16,17]. The db/db mouse model of T2DM exhibited cognitive dysfunction and emotional alterations, as well as increased IL-1β and tumor necrosis factor-α (TNF-α) levels in the plasma and hippocampus [18]. In addition, high glucose (HG) could activate the NLRP3 inflammasome in hippocampal neurons [19]. These findings strongly point to the NLRP3 inflammasome as an important factor affecting the development of DEP.

MCC950, a small-molecule NLRP3 inflammasome inhibitor [20], has been shown to ameliorate diabetic vascular endothelial dysfunction [21]. MCC950 is a diarylsulfonylurea-containing compound that was originally shown to block ASC oligomerization and inhibit NLRP3 inflammasome activation. MCC950 attenuated intracerebral hemorrhage against thrombin-induced NLRP3 inflammasome activation and cell apoptosis in microglia [22]. Moreover, MCC950 treatment obviously decreased neurological impairment, infarction volume, and neuronal apoptosis in a mouse model of ischemic stroke [23]. Dempsey and colleagues also identified that MCC950 could improve cognitive function in the APP/PS1 mice [24]. This was attributed to the inhibition of NLRP3 inflammasome activation and the reduction of Aβ accumulation in APP/PS1 mice.

Here, the present study aimed to investigate the effect of MCC950 in the db/db mouse model of T2DM and identify if it ameliorates DEP and explore the possible underlying pathways in vivo. Our results verify that the inhibition of NLRP3 inflammasome activation may be important to restricting the pathology of DEP.

## 2. Results

### 2.1. The Activation of NLRP3 Inflammasome in Hippocampus of Diabetic db/db Mice

**Figure 1:**
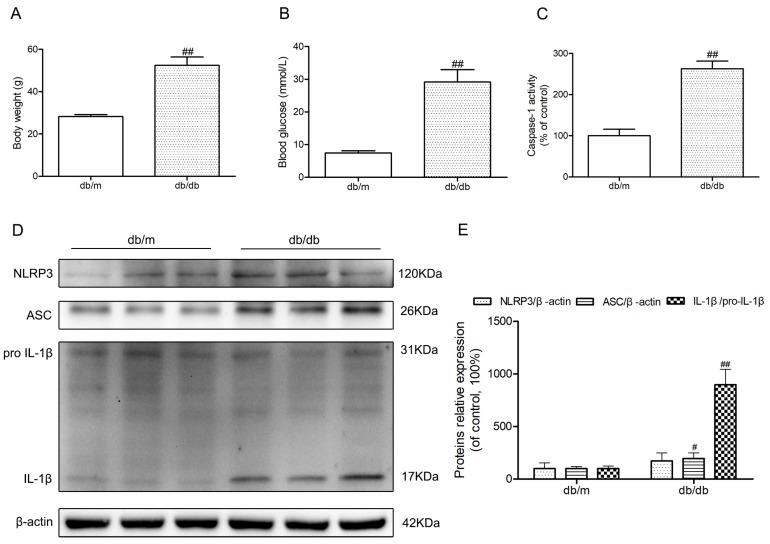
Activation of nucleotide-binding oligomerization domain-containing protein (NOD)-like receptor family, pyrin domain containing 3 (NLRP3) inflammasome in the hippocampus of db/db mice. (**A**) Body weights in mice of each group; (**B**) Fasting blood glucose in mice of each group; (**C**) Caspase-1 activity in the hippocampus of each group, as was reflected by the production of *p*-nitroaniline; (**D**) Western blot analysis of NLRP3 inflammasome-associated NLRP3, apoptosis-associated speck-like protein (ASC), interleukin (IL)-1β, and β-actin were performed in the hippocampus of each group; (**E**) The relative protein expression of NLRP3 to β-actin, ASC to β-actin, and IL-1β to pro-IL-1β are expressed in the bar graphs. All data are represented as mean ± SD (*n* = 3). ^##^ *p <* 0.01, ^#^ *p <* 0.05, compared with the db/m-group.

Body weights and fasting blood glucose were obviously increased in diabetic db/db mice compared to those of non-diabetic db/m mice (*p <* 0.01) (Figure 1A,B). Because of the possibility that the activation of NLRP3 inflammasome is involved in the neuroinflammatory component of DEP, we investigated NLRP3 inflammasome-related protein expressions in the hippocampus of diabetic db/db mice. As illustrated in Figure 1D, an obvious increase of active IL-1β in the db/db mice demonstrated that there may be NLRP3 inflammasome activation in the hippocampus of db/db mice (*p <* 0.01). Then, we detected the caspase-1 activity as well as NLRP3 and ASC expression levels. As shown in Figure 1C–E, NLRP3 and ASC expression levels and caspase-1 activity in hippocampal lysates from diabetic db/db mice were significantly increased compared to non-diabetic db/m mice. These results showed that there was NLRP3 inflammasome activation in the hippocampus of diabetic db/db mice.

### 2.2. Effect of MCC950 on Body Weights and Fasting Blood Glucose

**Figure 2:**
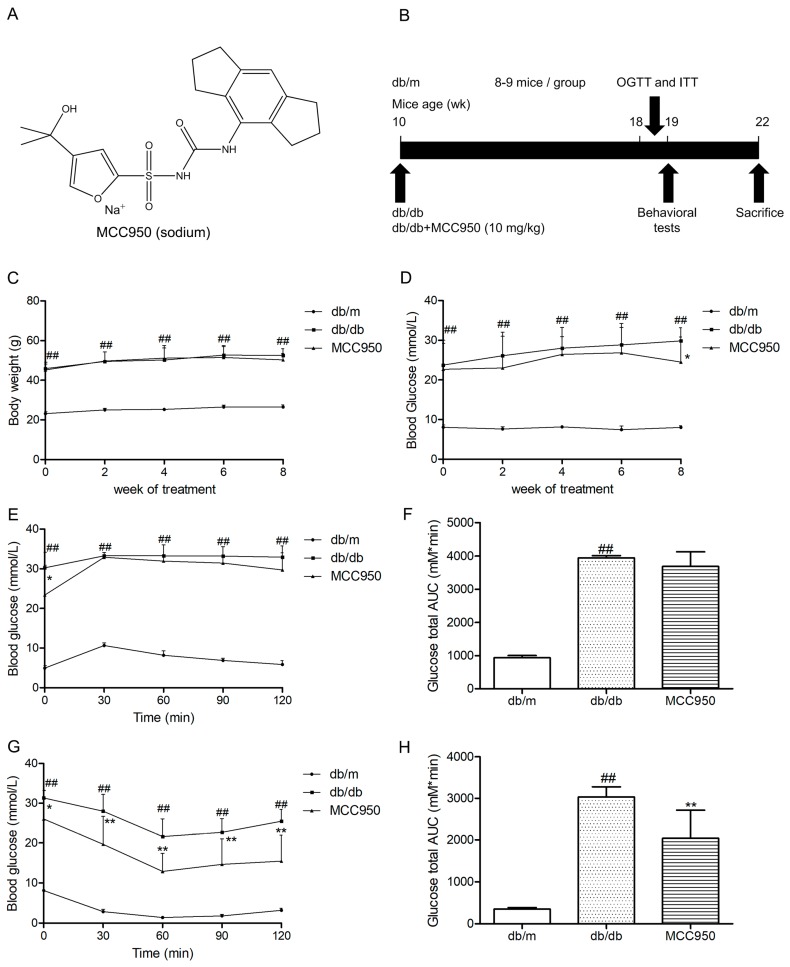
Effect of MCC950 on insulin resistance in db/db mice. (**A**) Chemical structure of MCC950 (sodium); (**B**) Schematic diagram showing the timeline scheme of the animal experiments in vivo; (**C**) Body weights in mice of each group during 8 weeks of treatment (*n* = 8–9 mice/group); (**D**) Fasting blood glucose in mice of each group during 8 weeks of treatment (*n* = 8–9 mice/group); (**E**) Curve of blood glucose levels in oral glucose tolerance tests (OGTTs; *n* = 8 mice/group); (**F**) Glucose total area under the curve (AUC) in OGTTs (*n* = 8 mice/group); (**G**) Curve of blood glucose levels in insulin tolerance tests (ITTs; *n* = 8 mice/group); (**H**) Glucose total AUC in ITTs (*n* = 8 mice/group). All data are represented as mean ± SD, ^##^ *p* < 0.01, compared with the db/m-group; ** *p* < 0.01, * *p* < 0.05, compared with the db/db-group.

As illustrated in Figure 2C,D, db/db mice showed an obvious increase in body weights and fasting blood glucose during the period of treatment compared to the non-diabetic db/m mice (*p* < 0.01). Treatment of MCC950 for 8 weeks could not obviously influence the body weights of db/db mice (*p* > 0.05) (Figure 2C). During 6 weeks of treatment, there were no obvious differences in fasting blood glucose between mice in the MCC950 group and vehicle-treated db/db mice (*p* > 0.05) (Figure 2D). However, after 8 weeks of treatment, the fasting blood glucose in the MCC950 group had an obvious decrease compared to the vehicle-treated db/db mice (*p* < 0.05).

### 2.3. Effect of MCC950 on Glucose Tolerance and Insulin Sensitivity

In oral glucose tolerance tests (OGTTs), the blood glucose at all time points and the glucose total area under the curve (AUC) in db/db mice were obviously higher than that of the db/m mice (*p* < 0.01) (Figure 2E). As shown in Figure 2F, there was no obvious difference in glucose total AUC between mice in the MCC950-treated group and vehicle-treated db/db mice until 8 weeks of intervention.

In insulin tolerance tests (ITTs), the MCC950 group showed a remarkable decrease in the blood glucose at all time points compared with the vehicle-treated diabetic db/db group (*p* < 0.05) (Figure 2G). Moreover, as shown in Figure 2H, treatment of MCC950 for 8 weeks obviously decreased the AUC of db/db mice in ITTs (*p* < 0.01).

### 2.4. MCC950 Reversed Anxiety- and Depression-Like Behavior in db/db Mice

**Figure 3:**
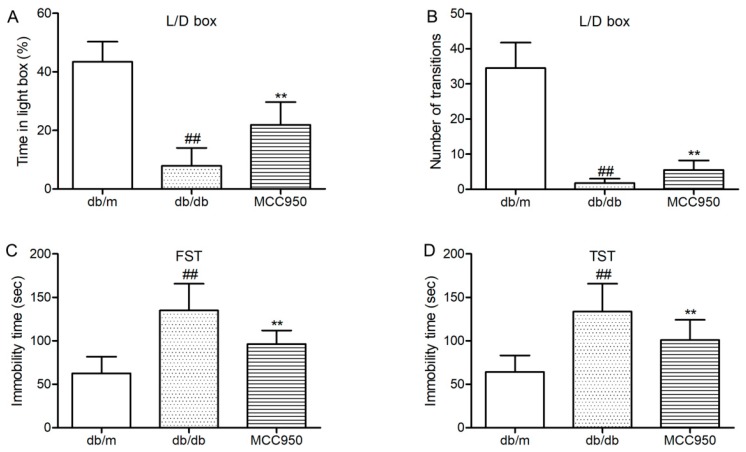
Effect of MCC950 on anxiety- and depression-like behaviors in db/db mice. (**A**) Percentage of time spent in lighted box of the light/dark (L/D) box; (**B**) Number of transition in the L/D box; (**C**) Immobility time in the forced swim test (FST); (**D**) Immobility time in the tail suspension test (TST). All data are represented as mean ± SD for eight mice in each group. ^##^ *p* < 0.01, compared with the db/m-group; ** *p* < 0.01, compared with the db/db-group.

First, we tested whether the anxiety-like behaviors of diabetic db/db could be inhibited by MCC950 treatment. Light/dark (L/D) box was used to assess the anxiety-like behaviors of db/db mice. As shown in Figure 3A,B, db/db mice showed few transitions between the light and dark boxes and spent proportionally less time in the light compartment of the L/D box. These results identified that db/db mice were more anxiogenic than non-diabetic mice. Interestingly, treatment with MCC950 could increase the time spent in the light box and the transition numbers between the light and dark boxes as compared to the vehicle-treated db/db mice (*p <* 0.01) (Figure 3A,B). These results suggested that MCC950 treatment ameliorated the anxiety-behaviors in db/db mice.

Next, we detected whether MCC950 could ameliorate depression-like behaviors in db/db mice. As shown in Figure 3C,D, forced swimming test (FST)and tail suspension test (TST) were performed to assess depression-like behaviors in db/db mice. In FST and TST (Figure 3C,D), db/db mice showed more immobility time as compared to the non-diabetic db/m mice (*p <* 0.01). As shown in Figure 3C,D, MCC950 treatment could significantly decrease the immobility time in FST and TST, which were considered as less depressive in db/db mice (*p <* 0.01).

These consistent data suggested that the inhibition of NLRPP3 inflammasome activation was beneficial to ameliorate anxiety- and depression-like behaviors in diabetic db/db mice.

### 2.5. MCC950 Ameliorated Cognitive Impairment in db/db Mice

**Figure 4:**
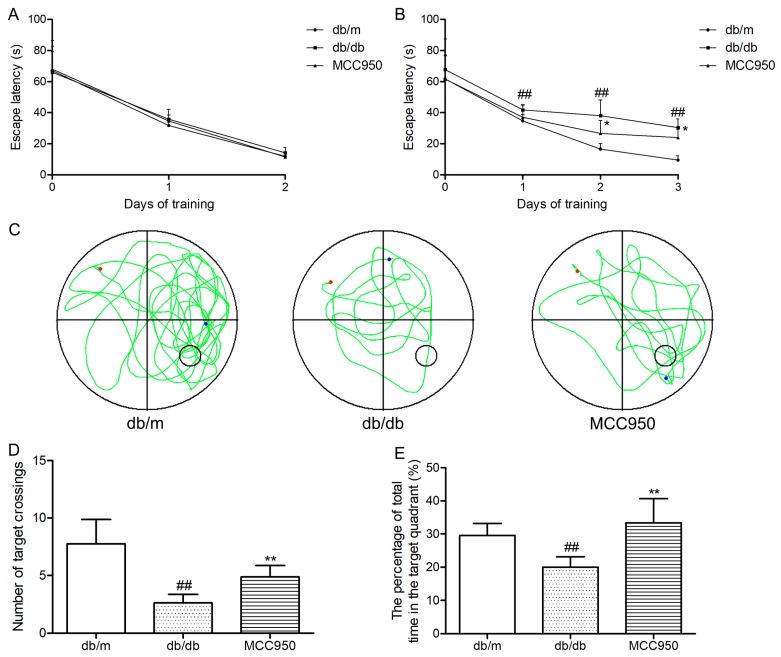
Effects of MCC950 on cognitive dysfunction in db/db mice. In the Morris water maze (MWM) test, day 0 means performance on the first trial, and subsequent points indicate the average of all daily trials. (**A**) Escape latency of the two-day visible-platform test;(**B**) Escape latency of the three-day hidden-platform test; (**C**) Representative swim paths during the probe test (the red dot represents the starting point, and the blue dot represents the end point); (**D**) Number of target crossings in the probe trial; (**E**) Percentage of total time spent in target quadrant in the probe trial. All data are represented as mean ± SD for eight mice in each group. ^##^ *p <* 0.01, compared with the db/m-group; ** *p <* 0.01, * *p <* 0.05, compared with the db/db-group.

First, we tested the escape latency of each mouse for 2 days in the visible-platform test of the Morris water maze (MWM). The aim of this test was to detect the vision and motivation between different groups. As shown in Figure 4A, mice in different groups showed similar escape latency (*p* > 0.05), indicating that there was no obvious difference among groups in vision and motivation.

Next, we tested the performance of each mouse for 3 days in the hidden-platform test of the MWM. As shown in Figure 4B, the escape latency in the db/db mice group significantly increased compared to that of the non-diabetic db/m mice (*p* < 0.01). Interestingly, MCC950 treatment obviously reduced the escape latency in db/db mice (*p* < 0.05).

After 5 days of training, the probe trial was performed on the sixth day. As shown in Figure 4C–E, the diabetic db/db mice exhibited an obvious reduction in the target crossing numbers and the percentage of total time in the target quadrant compared with those of the non-diabetic db/m mice (*p* < 0.01). In contrast, the MCC950 treatment group significantly increased the target crossing numbers and the percentage of total time in the target quadrant compared with the db/db group (*p* < 0.01). These data indicated that the inhibition of NLRP3 inflammasome activation could improve cognitive dysfunction in db/db mice.

### 2.6. MCC950 Inhibited Activation of the NLRP3 Inflammasome in the Hippocampus of db/db Mice

**Figure 5:**
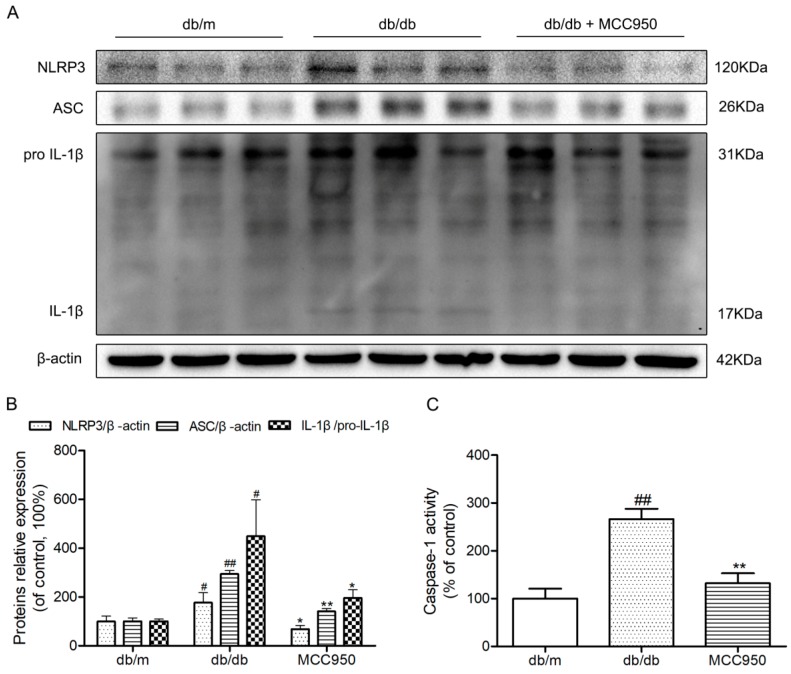
Effects of MCC950 on hippocampal NLRP3 inflammasome activation in db/db mice. (**A**) Western blot analysis of NLRP3 inflammasome-associated NLRP3, ASC, IL-1β, and β-actin were performed in the hippocampus of each group; (**B**) The relative protein expression of NLRP3 to β-actin, ASC to β-actin, and IL-1β to pro-IL-1β are expressed in the bar graphs; (**C**) Caspase-1 activity in the hippocampus of each group, as reflected by the production of *p*-nitroaniline. All data are represented as mean ± SD (*n* = 3). ^##^ *p* < 0.01, ^#^*p* < 0.05, compared with the db/m-group; ** *p* < 0.01, * *p* < 0.05, compared with the db/db-group.

To further investigate whether hippocampal NLRP3 inflammasome activation could be inhibited by MCC950 in db/db mice, a Western blot experiment was performed. Similar to our previous study, the NLRP3, ASC, and IL-1β expression levels and caspase-1 activity in the hippocampus of diabetic db/db mice were obviously increased compared with those in non-diabetic db/m mice (*p* < 0.05) (Figure 5). This once again identified that the hippocampus of db/db mice exhibited NLRP3 inflammasome activation. As expected, the increased NLRP3, ASC, and IL-1β expression levels and caspase-1 activity were successfully inhibited by 12 weeks of MCC950 treatment compared with the vehicle-treated db/db mice (*p* < 0.05). These results showed that MCC950 treatment could inhibit NLRP3 inflammasome activation in the hippocampus of db/db mice.

### 2.7. Effect of MCC950 on Insulin and IL-1β

**Figure 6:**
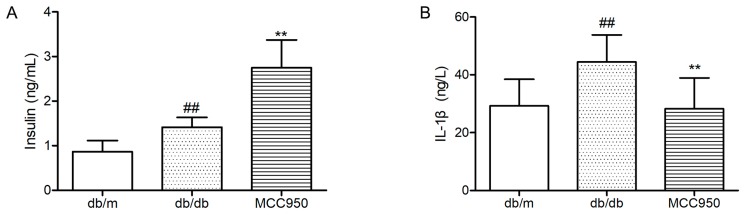
Effects of MCC950 on IL-1β, and insulin levels in plasma of db/db mice. (**A**) Levels of insulin in the plasma samples of mice; (**B**) Levels of IL-1β in the plasma samples of mice. All data are represented as mean ± SD (*n* = 8). ^##^ *p <* 0.01, compared with the db/m-group; ** *p <* 0.01, compared with the db/db-group.

As illustrated in Figure 6A, diabetic db/db mice showed an obviously higher level of plasma insulin than non-diabetic db/m mice (*p* < 0.01). Contrary to what we expected, the plasma level in MCC950-treated db/db mice was significantly higher than vehicle-treated db/db mice (*p* < 0.01). This result can probably be attributed to the protective effects of inhibiting NLRP3 inflammasome activation in pancreatic islets [25].

In the present study, we also detected the plasma IL-1β level to determine if peripheral inflammation was blocked by MCC950 treatment in db/db mice. First, we observed a significant increase of plasma IL-1β level in diabetic db/db mice compared with the non-diabetic db/m mice (*p <* 0.01) (Figure 6B). Next, we found that treatment with NLRP3 inflammasome inhibitor MCC950 for 12 weeks significantly decreased plasma IL-1β in db/db mice (*p <* 0.01).

### 2.8. Effect of MCC950 on TNF-α in Plasma and Hippocampus of db/db Mice

**Figure 7:**
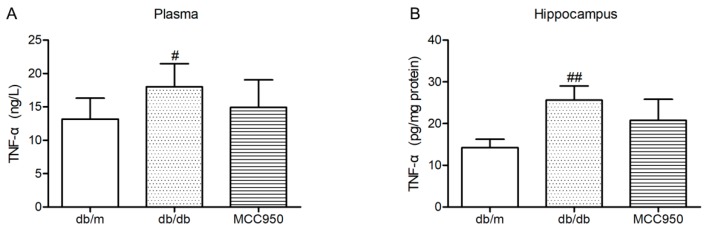
Effect of MCC950 on tumor necrosis factor-α (TNF-α) in the plasma and hippocampus of db/db mice. (**A**) Levels of TNF-α in the plasma samples of mice (*n* = 8 mice/group); (**B**) Levels of TNF-α in the hippocampus samples of mice (*n* = 3 mice/group). All data are represented as mean ± SD. ^##^ *p <* 0.01, ^#^ *p <* 0.05, compared with the db/m-group.

Similar to previous reports [18], the levels of TNF-α in the plasma and hippocampus of diabetic db/db mice were significantly increased compared with those of non-diabetic db/m mice (*p <* 0.05) (Figure 7). In the present study, MCC950 treatment failed to significantly decrease the levels of TNF-α in the plasma and hippocampus of db/db mice (*p* > 0.05).

## 3. Discussion

In the present study, our results demonstrated that the hippocampus of db/db mice exhibited NLRP3 inflammasome activation, indicating that the NLRP3 inflammasome was involved in the pathogenesis of DEP. Inhibition of hippocampal NLRP3 inflammasome activation by MCC950—a small-molecule inhibitor of the NLRP3 inflammasome—improved cognitive dysfunction as well as anxiety- and depression-like behaviors in db/db mice, thus offering an effective potential therapeutic approach for DEP treatment.

Similar to previous reports [26,27,28], our study suggested that the db/db murine model of T2DM exhibited symptoms like obesity, hyperglycemia, hyperinsulinemia, cognitive dysfunction, anxiety- and depression-like behaviors, indicating that db/db mice could be used as an experimental model of DEP. The pathogenesis of DEP is complicated and not absolutely clear—a fairly explicit viewpoint of which is that inflammation is the major influencing factor of DEP [1,18]. IL-1β, an important pro-inflammatory cytokine, was obviously higher in the hippocampus of db/db mice than that of the control non-diabetic mice in previous reports [18,27]. In the present study, the active IL-1β level in the hippocampus of diabetic db/db mice significantly increased as compared to the non-diabetic db/m mice (*p <* 0.01). These findings demonstrated that the NLRP3 inflammasome might be activated in the hippocampus of db/db mice.

The NLRP3 inflammasome is a vital mediator of IL-1β production [29]. NLRP3 has a tripartite structure consisting of a nucleotide-binding domain (NBD), a pyrin domain (PYD), and a leucine-rich-repeat (LRR) domain [30]. Upon activation, NLRP3 can interact with the adaptor protein ASC (which has a caspase activation and recruitment domain, CARD) to recruit pro-caspase-1, thus forming the NLRP3 inflammasome [31]. Once activated, the NLRP3 inflammasome induces caspase-1 activation by processing pro-IL-1β into biologically mature IL-1β. In this study, higher expression levels of NLRP3 and ASC and activity of caspase-1 were observed in the hippocampus of db/db mice, while there was obviously decreased NLRP3, ASC expression level, and caspase-1 activity in the hippocampus of db/m mice, indicating that the NLRP3 inflammasome was involved in the development of DEP.

Recent studies demonstrated that inhibiting the hippocampal NLRP3 inflammasome activation could ameliorate cognitive dysfunction and depression in mice. Heneka and colleagues showed that APP/PS1/NLRP3^−/−^ and APP/PS1/Casp-1^−/−^ mice were largely protected from memory impairment in APP/PS1 mice [14]. Moreover, inhibition of the NLRP3 inflammasome activation could reduce hippocampal IL-1β expression level and obviously improved the anxiety- and depressive-like behaviors in mice [16,17]. Therefore, inhibiting hippocampal NLRP3 inflammasome activation may be a potential therapeutic approach to the treatment of AD, depression, and anxiety.

T2DM is a metabolic disorder with chronic low-grade inflammation. Numerous studies showed that the NLRP3 inflammasome was a critical regulator involved in the development of T2DM and its complications [32,33]. Studies identified that inhibiting NLRP3 inflammasome activation could ameliorate diabetic cardiomyopathy [34], diabetic retinopathy [35], diabetic nephropathy [36], and diabetic vascular endothelial dysfunction [21]. Furthermore, the reduction of NLRP3 inflammasome expression has been associated with improved insulin sensitivity in obese T2DM patients [37].

To explore if inhibiting NLRP3 inflammasome activation can ameliorate DEP, we used the NLRP3 inflammasome inhibitor MCC950 in diabetic db/db mice and detected cognitive and emotional alterations, as well as the NLRP3, ASC, and IL-1β expression levels and caspase-1 activity in the hippocampus, and compared with those of the vehicle-treated mice. In the present study, treatment with MCC950 (10 mg/kg) was efficacious in ameliorating anxiety-like behaviors in L/D box, depression-like behaviors in TST and FST, cognitive disorders in MWM test, as well as decreasing hippocampal NLRP3, ASC, and IL-1β expression levels and caspase-1 activity in db/db mice. Additionally, the plasma IL-1β level was significantly reduced after 12 weeks treatment of MCC950. Interestingly, the present study showed that MCC950 treatment failed to inhibit the increase of TNF-α level in plasma and hippocampus of db/db mice. The effect of MCC950 treatment on decreasing IL-1β level in db/db mice was stronger than that of TNF-α, demonstrating that the inhibition of IL-1β secretion was specific. We also observed that MCC950 treatment could improve insulin sensitivity in db/db mice, which might result from inhibiting NLRP3 inflammasome activation [38]. These data indicated that the inhibition of NLRP3 inflammasome activation was a novel therapeutic approach to ameliorate DEP.

However, our study also has some limitations; for example, the experimental animal models we chose are simple, and we should use a variety of diabetes animal models for further investigation of the role of the NLRP3 inflammasome in DEP.

## 4. Materials and Methods

### 4.1. Reagent and Materials

MCC950 (sodium) (molecular weight = 426.46, molecular formula: C_20_H_23_N_2_NaO_5_S, CAS NO: 256373-96-3, purity > 99.40%) was obtained from MedChem Express (Shanghai, China). Normal saline was produced from Shandong Hualu Pharmaceutical Co., Ltd. (Liaocheng, China). Insulin injection was supplied from WanbangBioPharma (Xuzhou, China). The ELISA kit for measuring IL-1β, the caspase-1 activity assay kit, and the Bradford protein assay kit were purchased from Beyotime Institute of Biotechnology (Beijing, China). The ELISA kit for determining the mouse insulin level was obtained from ALPCO (Salem, MA, USA). The ELISA kit for measuring TNF-α was purchased from DAKEWEI (Shenzhen, China). Primary antibody against NLRP3 (ab214185) was obtained from Abcam (Cambridge, UK). Primary antibodies against ASC (sc-514414) and IL-1β (sc-7884) were purchased from Santa Cruz Biotechnology (Santa Cruz, CA, USA). The peroxidase-conjugated secondary antibodies of goat anti-rabbit IgG (ZB-2301) and goat anti-mouse IgG (ZB-2305) were purchased from ZSJQ-BIO (Beijing, China). Primary antibody against β-actin (CW0096) and kits for tissue protein extraction, protease inhibitor, bicinchoninic acid (BCA) protein quantization, and enhanced chemiluminescence regeat were bought from CoWin Biosciences (Beijing, China).

### 4.2. Animals and Groups

The animal experiments were conducted in two steps. The first step was to identify whether NLRP3 inflammasome activation existed in the hippocampus of db/db mice. Male 22-week old diabetic mice (BKS.Cg-Dock7^m +/+^ Lepr^db^/Nju, db/db, *n* = 3) and age-matched non-diabetic mice (db/m, *n* = 3) were used in this experiment. At the second step, the effects of NLRP3 inflammasome inhibitor MCC950 on behavior alterations of diabetic db/db mice were investigated. Male 9-week old diabetic db/db mice and age-matched non-diabetic mice (db/m, *n* = 8) were used in this experiment. The mice were purchased from the Model Animal Research Center of Nanjing University (Nanjing, China). The mice were housed under controlled temperature (22 ± 2 °C) and humidity (60 ± 10%) with a 12 h light–dark cycle. The animals were allowed free access to standard diet and water. All experimental procedures were in accordance with the Animal Ethics Committee of the Chinese Academy of Medical Sciences and Peking Union Medical College (SCXK 2014-0001).

### 4.3. Experimental Protocol

After one week of adaption, all diabetic db/db mice were randomly divided into two groups: model control group (db/db), and db/db + MCC950 group (MCC950); *n* = 9 per group. The non-diabetic lean mice (*n* = 8) were used as control group (db/m). All the mice received intraperitoneal injections of sterile phosphate-buffered saline (PBS; db/m and db/db groups) or MCC950 (10 mg/kg in PBS, MCC950 group) every day for 12 weeks, and the behavioral tests were performed after 9 weeks of treatment. Body weights and fasting blood glucose were measured every 2 weeks. After 6 h of fasting food, body weights and blood glucose were detected by an electronic weighing scale (YP2002N, Shanghai Jinghai Instrument Co., Ltd., Shanghai, China) or a portable glucometer (Roche Group, Basel, Switzerland).

### 4.4. Oral Glucose Tolerance Test and Insulin Tolerance Test

Oral glucose tolerance test (OGTT) and insulin tolerance test (ITT) were conducted as previously reported with little modification [39]. After an overnight fast, OGTT was performed by oral administration of glucose solution (1 g/kg). After 6 h of fasting food, ITT was performed by intraperitoneal injection of insulin (0.75 U/Kg) in sterile normal saline. The levels of blood glucose were monitored at 0, 30, 60, 90, and 120 min after treatment using a portable glucometer.

### 4.5. Behavioral Testing

Except for the tail suspension and forced swim tests, a video computerized tracking system (Super Maze, Shanghai Xinruan Information Technology Co., Ltd., Shanghai, China) was used to record the behaviors of mice. All experiments were performed at 9:00 a.m.–5:00 p.m. under conditions of dim light and low noise.

#### 4.5.1. Light/Dark Box Test

The light/dark (L/D) box test was performed as previously reported with few modifications [27]. The L/D box test was used to evaluate anxiety-like behavior in db/db mice. The apparatus contained two boxes: one white acryl box (17 × 18 × 19 cm) with bright light and another black acryl box (17 × 18 × 19 cm) without light. There was an opening (5 × 7 cm) between the two boxes. After placing the mouse in the black box, the opening was blocked for 5 s. Then, the mouse was allowed to freely explore for 10 min in the apparatus. Parameters measured to assess anxiety-like behavior were the transition numbers between the two boxes and time spent in the light box. The box was thoroughly cleaned using 75% alcohol before each use to remove odor cues.

#### 4.5.2. Tail Suspension Test

The tail suspension test (TST) was performed following the previous method with slight modifications [3]. Each mouse was suspended vertically by the tail on the top of an opaque box (30 × 30 × 30 cm). Each mouse performed this test for 6 min. The immobility time was computed for the last 4 min in TST. Except for movements caused by respiration, immobility was defined as the absence of any movements.

#### 4.5.3. Forced Swim Test

The forced swim test (FST) was performed as previously reported with slight modifications [3]. Mice were individually placed into a beaker (20 cm height × 13 cm diameter) with 25 °C water 15 cm deep. The mouse could not touch the bottom to support itself. One camera was placed above the cylinder to videotape the behavior of each mouse in water. The immobility time was measured for the last 4 min in FST. Immobility was defined as floating in the water without struggling and only keeping itself above the water with necessary movements.

#### 4.5.4. Morris water maze (MWM) Test

The Morris water maze (MWM) test was performed following the method described previously [28]. The test included the 2-day visible-platform training, the 3-day hidden-platform training, and a probe trial on day 6. The water maze equipment consisted of a black circular pool (100 cm in diameter, 50 cm in height), a platform (9 cm in diameter), and a computer equipped with a recording system. The water in the pool was maintained at 25 ± 1 °C. The pool was spatially divided into four imaginary quadrants, and the platform was placed 1 cm below the water surface in the center of one quadrant without movement during the experiment. In the visible-platform training, a small flag (5 cm in height) was placed on the platform. In the hidden-platform training, the flag was removed. For each daily trial, each mouse performed four trials with a 1 h interval. The escape latency data was recorded. If the mouse failed to find the platform within 90 s, the trial was ended and the mouse was guided to the platform for 10 s, and its escape latency data was recorded as 90 s. On the 6th day, the probe trial was performed. Each mouse was allowed to swim freely in the pool for 90 s without the platform. The numbers of each mouse crossing through the original platform position and the time spent in the target quadrant were recorded.

### 4.6. Tissue Collection

After the behavioral experiments, blood samples of mice were collected into EDTA (10%)-coated chilled tubes. The samples were centrifuged at 3000× *g* for 10 min at 4 °C, and plasma samples were collected for further measurement. Mice were perfused with cold PBS through the ascending aorta. Each mouse was decapitated, and hippocampus and cortex were rapidly dissected. The tissue samples were frozen in liquid nitrogen and then stored at −80 °C for further experiments.

### 4.7. Insulin, IL-1β, and TNF-α Levels Analysis

The levels of insulin, IL-1β, and TNF-α were measured by ELISA kits following the manufacturer’s instructions.

### 4.8. Measurement of Caspase-1 Activity

The activity of caspase-1 in the hippocampus was detected by commercial assay kits according to the manufacturer’s instructions. The analysis of caspase-1 activity was based on the cleavage of the Ac-YVAD-*p*NA (acetyl-Tyr-Val-Ala-Asp *p*-nitroanilide) substrate into the yellow *p*-nitroaniline (*p*NA), which has strong absorbance at 405 nm. The tissues were homogenized, and the tissue homogenates were centrifuged at 16,000× *g* for 15 min at 4 °C. The concentrations of total proteins were measured by a Bradford assay kit. The hippocampus supernatants were incubated with 10 μL Ac-YVAD-*p*NA for 2 h at 37 °C in dark. The absorbance values were measured by a microplate reader (Infinite M1000, Tecan, Sunrise, Austria) at a wavelength of 405 nm. The caspase-1 activity was calculated by the standard curve of *p*NA. The results are presented as relative activity of caspase-1 of the db/m group.

### 4.9. Western Blot Analysis

Western blot was performed as previously reported [40]. The sample tissues were weighed and homogenized in ice-cold lysis buffer (1:100 inhibitor proteases and phosphatases cocktail). The total protein concentration was detected with bicinchoninic acid (BCA) assay. The samples were run on an SDS-PAGE gel and transferred onto nitrocellulose membranes. The membranes were treated with primary and secondary antibodies. The primary antibodies NLRP3 (1:1000), ASC (1:200), IL-1β (1:200), and β-actin (1:1000) were used for Western blot analysis. The signals were visualized by an enhanced chemiluminescence reagent.

### 4.10. Statistical Analysis

Data were expressed as means ± standard deviation (SD). All results were analyzed by a one-way analysis of variance (ANOVA) based on Student’s two-tailed unpaired *t*-test. The *p*-values less than 0.05 were considered to be statistically significant.

## 5. Conclusions

In conclusion, our present study reveals that the NLRP3 inflammasome is involved in DEP via regulating the production and maturation of IL-1β in the hippocampus. Furthermore, the NLRP3 inflammasome inhibitor MCC950 ameliorates cognitive disorders as well as anxiety- and depression-like behaviors in db/db mice, which is associated with the inhibition of hippocampal NLRP3 inflammasome activation. Our findings indicate that inhibiting hippocampalNLRP3 inflammasome activation may be a therapeutic approach to alleviating DEP in the future.
